# Impact of rural address and distance from clinic on depression outcomes within a primary care medical home practice

**DOI:** 10.1186/s12875-019-1015-7

**Published:** 2019-09-05

**Authors:** Hailon Wong, Kyle Moore, Kurt B. Angstman, Gregory M. Garrison

**Affiliations:** Department of Family Medicine, Mayo Clinic, 200 First Street SW, Rochester, MN 55905 USA

**Keywords:** Depression, Primary care, Rural-urban disparity, Collaborative care management

## Abstract

**Background:**

Depression is the second leading cause of death among young adults and a major cause of disability worldwide. Some studies suggest a disparity between rural and urban outcomes for depression. Collaborative Care Management (CCM) is effective in improving recovery from depression, but its effect within rural and urban populations has not been studied.

**Methods:**

A retrospective cohort study of 3870 patients diagnosed with depression in a multi-site primary care practice that provided optional, free CCM was conducted. US Census data classified patients as living in an Urban Area, Urban Cluster, or Rural area and the distance they resided from their primary care clinic was calculated. Baseline demographics, clinical data, and standardized psychiatric assessments were collected. Six month Patient Health Questionnaire (PHQ 9) scores were used to judge remission (PHQ9 < 5) or Persistent Depressive Symptoms (PDS) (PHQ9 ≥ 10) in a multivariate model with interaction terms.

**Results:**

Rural patients had improved adjusted odds of remission (AOR = 2.8) and PDS (AOR = 0.36) compared to urban area patients. The natural logarithm transformed distance to primary care clinic was significant for rural patients resulting in a lower odds of remission and increased odds of PDS with increasing distance from clinic. The marginal probability of remission or PDS for rural patients equaled that of urban area patients at a distance of 34 or 40 km respectively. Distance did not have an effect for urban cluster or urban area patients nor did distance interact with CCM.

**Conclusion:**

Residing in a rural area had a beneficial effect on the recovery from depression. However this effect declined with increasing distance from the primary care clinic perhaps related to greater social isolation or difficulty accessing care. This distance effect was not seen for urban area or urban cluster patients. CCM was universally beneficial and did not interact with distance.

## Background

Depression affects 8.1% of Americans and is the single largest contributor to non-fatal health loss throughout the world [1, 2]. It has been associated with decreased functional status, quality of life, and income along with substantial societal costs in the form of lost work productivity [3, 4]. It is a chronic condition characterized by high rates of recurrence and relapse and is often comorbid with other chronic conditions [5].

Some studies have shown that depression is slightly more common in rural vs urban settings, although these differences were no longer present after controlling for patient characteristics [6]. Others have found no overall difference in prevalence between rural and urban areas, but higher prevalence in urban areas when potential confounders were controlled [7]. Several authors have also noted that outcomes including quality of life and treatment effectiveness are worse in rural vs urban settings [8, 9]. Possible contributors to this rural-urban disparity include lack of access to medical care, greater distance to care, stigma, and relative lack of recreational resources [9].

The United States (US) Census Bureau classifies specific geographic areas as urban areas (population 50,000 or greater) or urban clusters (population 2500 to 50,000) with the remaining land mass designated as rural [10]. Rural areas make up 97% of US land mass but contain just 19.3% of the population [11].

Treatment of depression is highly cost-effective but underutilized. Among depression treatment options, the collaborative care model (CCM) has emerged as a promising and more effective approach to the treatment of depression [12]. CCM utilizes a team consisting of trained registered nurse care coordinators, behavioral health specialists, and primary care physicians [12]. Several authors have shown that collaborative care shortens the time to depression remission and sustains remission for a longer duration [13, 14].

Few studies have examined the relationship between the collaborative care model and the rural-urban depression outcome disparity. The objective of this study was to compare depression outcomes in rural vs urban settings while accounting for the known differences between CCM and usual care within a large Southeast Minnesota primary care practice. We hypothesized that among primary care patients treated for depression, outcomes would be worse among patients from rural settings irrespective of treatment type but that this effect would be mitigated for those treated with CCM.

## Methods

A retrospective cohort study of patients diagnosed with depression was conducted to determine the effects of patient location on depression treatment outcomes. All adult patients (age ≥ 18) diagnosed with major depression (ICD9 codes 296.2 to 296.3) or dysthymia (ICD-9 code 300.4) with an initial Patient Health Questionnaire- 9 (PHQ-9) [15] score ≥ 10 who were treated at any of five primary care clinics located in Rochester, Minnesota and surrounding communities were eligible for inclusion in the study. The only exclusion criterion was a diagnosis of bipolar disorder (ICD9 codes 296.4 to 296.8). The study was reviewed and approved by the Mayo Clinic Institutional Review Board.

### Cohort

**Fig. 1:**
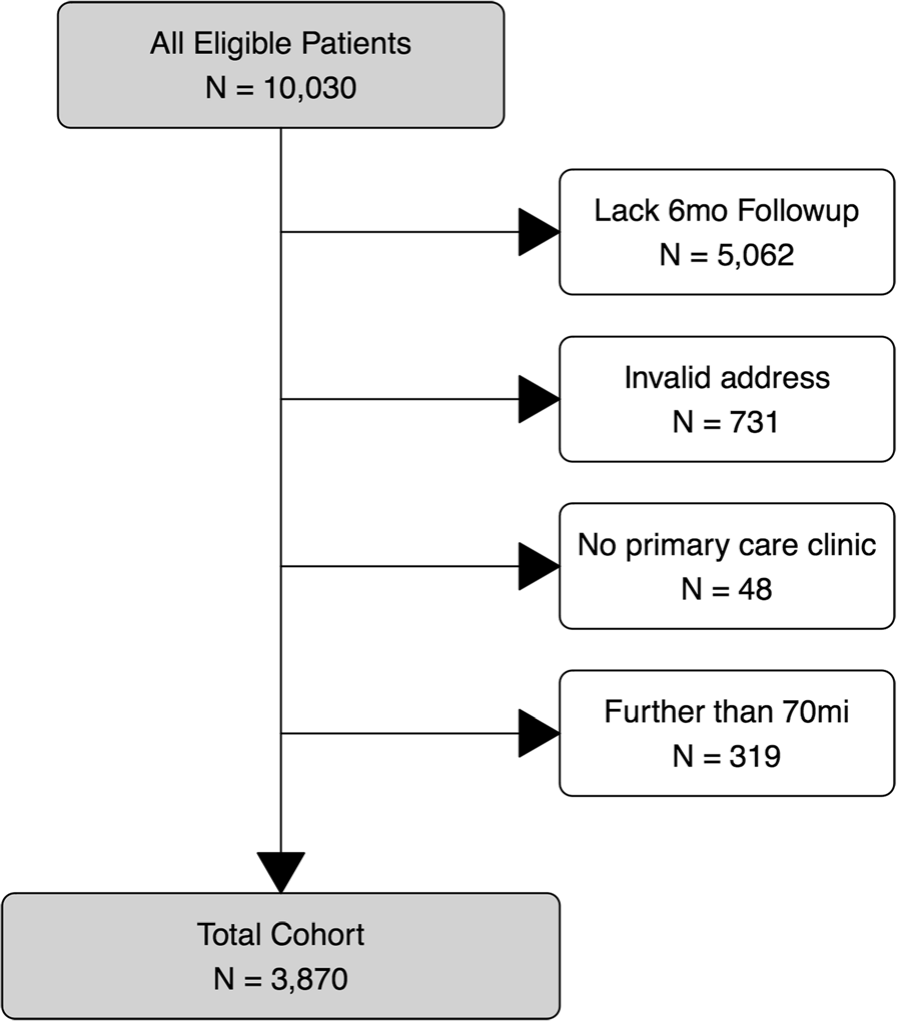
Cohort selection

During the study period from March 1, 2008 to December 31, 2015, there were 10,030 patients who met the criteria and gave permission to review their medical record for research purposes. Clinical guidelines and government quality metrics define remission of depression based upon PHQ-9 values 6 months following diagnosis [16–18]. 4968 patients had 6 month followup PHQ-9 scores recorded in the medical record, enabling outcome measurement and thus formed the cohort. Patients whose address could not be mapped to the 2010 US Census dataset (*n* = 731) or who lacked a clearly identified primary care clinic (*n* = 48) were excluded from analysis. Some patients had addresses that placed them far outside the typical catchment area for a primary care clinic, perhaps because they spent winters in a second home or had mail sent to a relative. Thus, any patient address farther than 70 miles (112.7 km) from their primary care clinic was also excluded (*n* = 319). The remaining 3870 patients were analyzed. Figure 1 provides a flow diagram of the cohort selection.

### Setting

Each clinic provided Collaborative Care Management (CCM) for depression within a patient centered medical home practice. The CCM model has been described in detail previously; briefly it supports the primary care clinician in caring for depressed patients with an electronic registry, specially trained registered nurse care managers, the use of established clinical guidelines, and an integrated behavioral health team consisting of psychologists, social workers, clinical nurse specialists, and a consulting psychiatrist [12, 17, 19]. The care managers, social workers, and psychologists are located in each clinic to maintain face-to-face, telephone, or electronic contact with CCM patients. They utilize practice guidelines and consultation with a psychiatrist to assist the primary care clinician in caring for depressed patients by providing counseling, medication advice, and frequent followup. During the study time period, CCM was an optional service provided at no extra cost to the patient. Patients were free to choose CCM or usual care based upon their preferences. Compared to usual care, CCM has been shown to improve depression outcomes in our practice [14].

### Data collection

For the remaining 3870 patients, data was collected including demographics (age, gender, race, marital status, smoking status), diagnosis (initial vs. recurrent depression; initial PHQ-9, Generalized Anxiety Disorder- 7 (GAD-7) [20] and Mood Disorder Questionnaire (MDQ) [21] scores), treatment (CCM enrollment, 6 month PHQ-9 score), and location (address, primary care site). Two outcome variables were constructed; Remission defined by a 6 month PHQ-9 < 5 and Persistent Depressive Symptoms (PDS) defined by a 6 month PHQ-9 ≥ 10 [22, 23]. The patient’s address was mapped to a latitude and longitude using tools from the US Census Bureau and geocoding software [24]. Their address was classified as an urban area (population > = 50,000), urban cluster (population 2500–50,000), or rural (all other areas) location based upon the 2010 US Census dataset Legal/Statistical Area Descriptions (LSAD) [25]. Distance from the patient’s address to their primary care clinic was calculated in kilometers using the Haversine formula [26]. The other independent variables were included to adjust depression outcomes as they are known to be significant [17, 27].

### Analysis

Statistical analysis was carried out using R 3.6.1 [28]. Data was grouped by US Census LSAD address classification (urban area, urban cluster, or rural). Continuous independent variables were summarized by mean and standard deviation. Frequencies were calculated for categorical independent variables. Comparison between address classification was performed by Chi-Square tests for categorical variables or ANOVA for continuous variables with *p*-values less than 0.05 considered significant.

In order to assess our hypothesis, a multivariate logistic regression model was constructed for both dependent variables (Remission and PDS) using the independent variables, the natural logarithm of distance to primary care site, address classification, and interaction terms. Interaction terms were included because it was hypothesized that distance to primary care site may have a different impact in rural areas as compared to urban areas. Distance is by nature a positive number and is highly right skewed; therefore it was transformed by taking the natural logarithm to produce a more normal distribution that maintains only positive predicted transformed values. Missing GAD-7 scores were imputed by linear regression on the demographic variables using the missing-at-random (MAR) assumption for 1156 patients. Because of the significant number of patients with missing 6 month outcomes, we also performed a worst case analysis on all eligible patients assuming lack of remission and continued PDS for those with missing 6 month outcomes. 95% confidence intervals were calculated for all coefficients and odds ratios were reported. Variables with Pr(>|z|) less than 0.05 were considered significant.

## Results

**Fig. 2:**
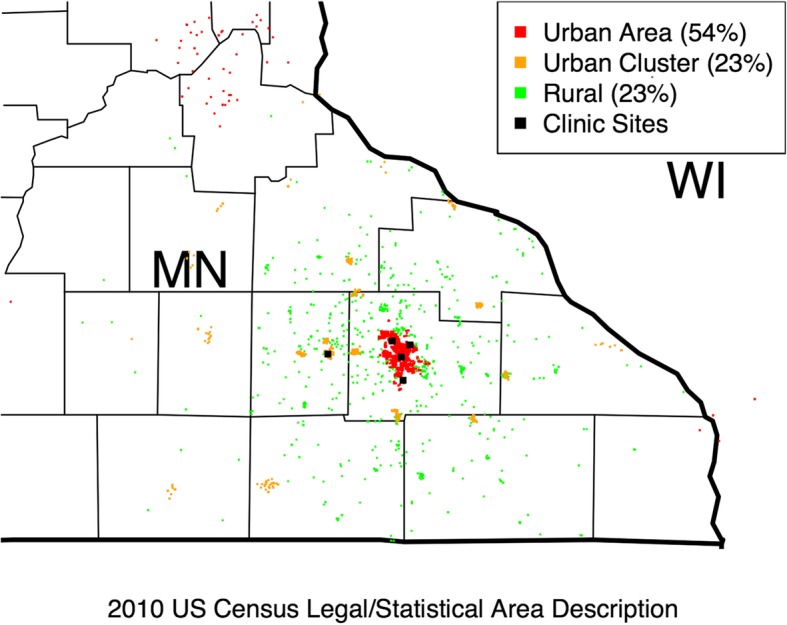
Location and classification of depressed patients

A total of 3870 patients with depression were analyzed, 54.0% resided in urban areas, 22.9% in urban clusters, and 23.1% in rural areas as shown on the map of patient locations in Fig. 2. As summarized in Table 1, rural patients were older, were more likely to be white, and more likely to be married than those in urban areas or urban clusters. The highest smoking prevalence was found among urban cluster patients. The prevalence of recurrent depression, initial PHQ-9, GAD-7, and MDQ scores were not different among the groups studied. A similar percentage of patients chose to enroll in CCM from each group. As expected based upon the location of the clinics, there were significant differences in the rural/urban population mix they served. To control for this, clinic site was included as an independent variable in the multivariate analysis. Rural and urban cluster patients were located farther from their primary care clinics than urban area patients. Rural patients had the highest incidence of remission and the lowest incidence of PDS, whereas urban cluster patients had the lowest incidence of remission and the highest incidence of PDS.

**Table 1:** Characteristics of depressed patients by address classification

	Urban Area	Urban Cluster	Rural	*p*-value
*N* (%)	2088 (54.0%)	885 (22.9%)	897 (23.1%)	–
Age, mean (SD), yrs	43.2 (16.8)	41.6 (15.4)	44.2 (16.4)	0.003
Gender, % Female	72.8%	74.4%	70.9%	0.260
Race, % White	90.2%	96.6%	97.8%	< 0.001
Married, %	43.4%	46.4%	56.1%	< 0.001
Recurrent Depression, %	44.5%	40.5%	42.5%	0.115
Initial PHQ-9, mean (SD)	15.5 (4.1)	15.2 (4.1)	15.3 (4.2)	0.294
GAD-7, mean (SD)	11.6 (5.3)	11.7 (5.4)	11.2 (5.5)	0.190
MDQ positive	12.3%	14.4%	13.4%	0.279
Smoker	13.8%	22.3%	13.3%	< 0.001
Care Management, %	80.0%	78.3%	79.9%	0.560
Clinic				< 0.001
A	63.7%	16.3%	20.0%	
B	15.9%	55.0%	29.2%	
C	62.5%	14.1%	23.4%	
D	55.0%	22.5%	22.6%	
E	60.4%	13.9%	25.6%	
Distance from Clinic, mean (SD), km	14.7 (17.4)	28.9 (16.6)	32.6 (18.5)	< 0.001
Remission @ 6mo, %	45.5%	43.1%	48.8%	0.048
PDS @ 6mo, %	29.0%	32.4%	25.8%	0.008

Address Classification based upon 2010 United States Census Legal Statistical Area Description

*p*-values < 0.05 considered significant

### Remission

**Fig. 3:**
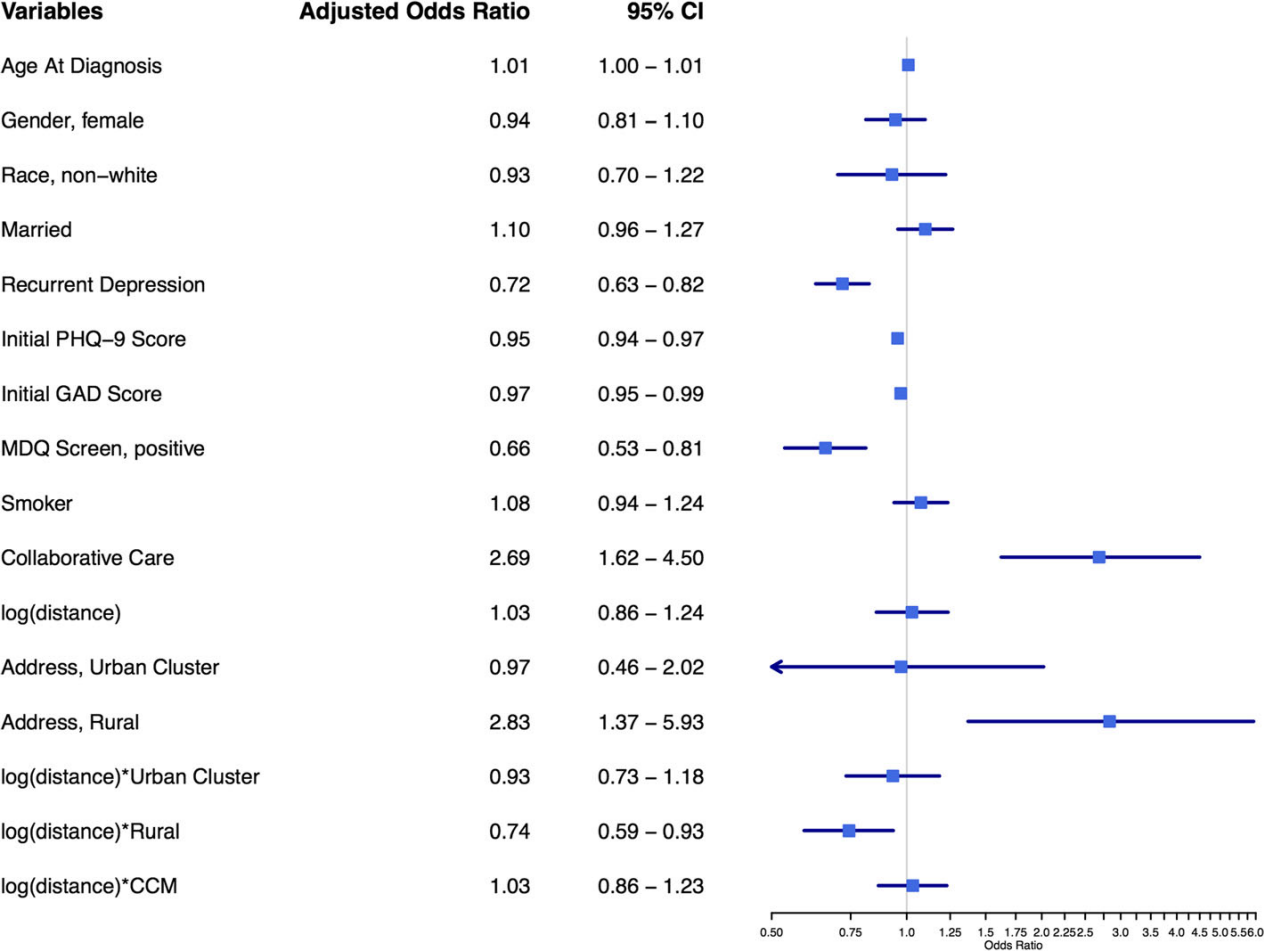
Multivariate Odds Ratios for Remission (PHQ-9 <5) at six months, controlling for clinical site. Baseline probability group is an unmarried, white, nonsmoking, male with initial depression who lives in an urban area and chooses UC. The final three lines are interaction terms between the variables separated by asterisks

**Fig. 4:**
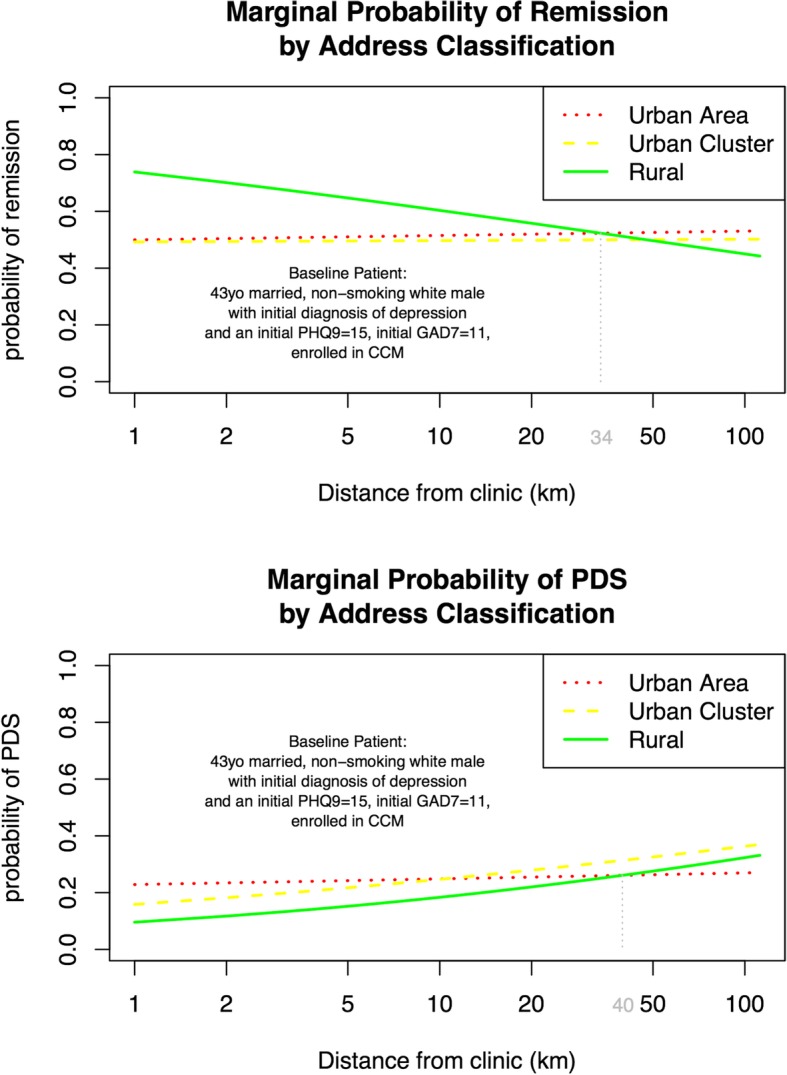
Marginal Probability vs. Distance for each LSAD Group. Abbreviations used: LSAD = US Census Legal/Statistical Area Description; PDS = Persistent Depressive Symptoms; CCM = Collaborative Care Management

As expected based on prior work, multivariate analysis of remission revealed that most DOC-6 components (age, recurrent depression, initial PHQ-9 score, GAD-7 score, and MDQ), smoking status, and enrollment in CCM were all significantly associated with adjusted remission odds as shown in Fig. 3 [14, 17, 27, 29, 30]. Rural patients had improved adjusted odds of remission (AOR = 2.8, 95%CI 1.4–5.9) compared to urban area patients while urban cluster patients were not different. The natural logarithm transformed distance to primary care clinic was associated with reduced remission for rural patients (β = − 0.292, AOR 0.75, 95%CI 0.60–0.94). However, this was not true for urban areas or urban clusters as shown by the interaction terms. Because interpretation of adjusted odds ratios for interaction terms containing transformed continuous and dichotomous variables is difficult to conceptualize, Fig. 4 was created to show the marginal probability of remission in a hypothetical baseline patient for each address classification at varying distances. The probability of remission for rural patients was higher but declined with increasing distance from clinic, whereas the probability of remission for urban area and urban cluster patients was approximately constant over distance when other variables were held constant. At approximately 34 km from the primary care clinic, the probability of remission for a rural patient is similar to an urban patients with the same risk factors. Farther out than 34 km, rural patients showed a lower probability of remission. Distance did not interact with CCM when other variables were controlled.

### Persistent depressive symptoms

**Fig. 5:**
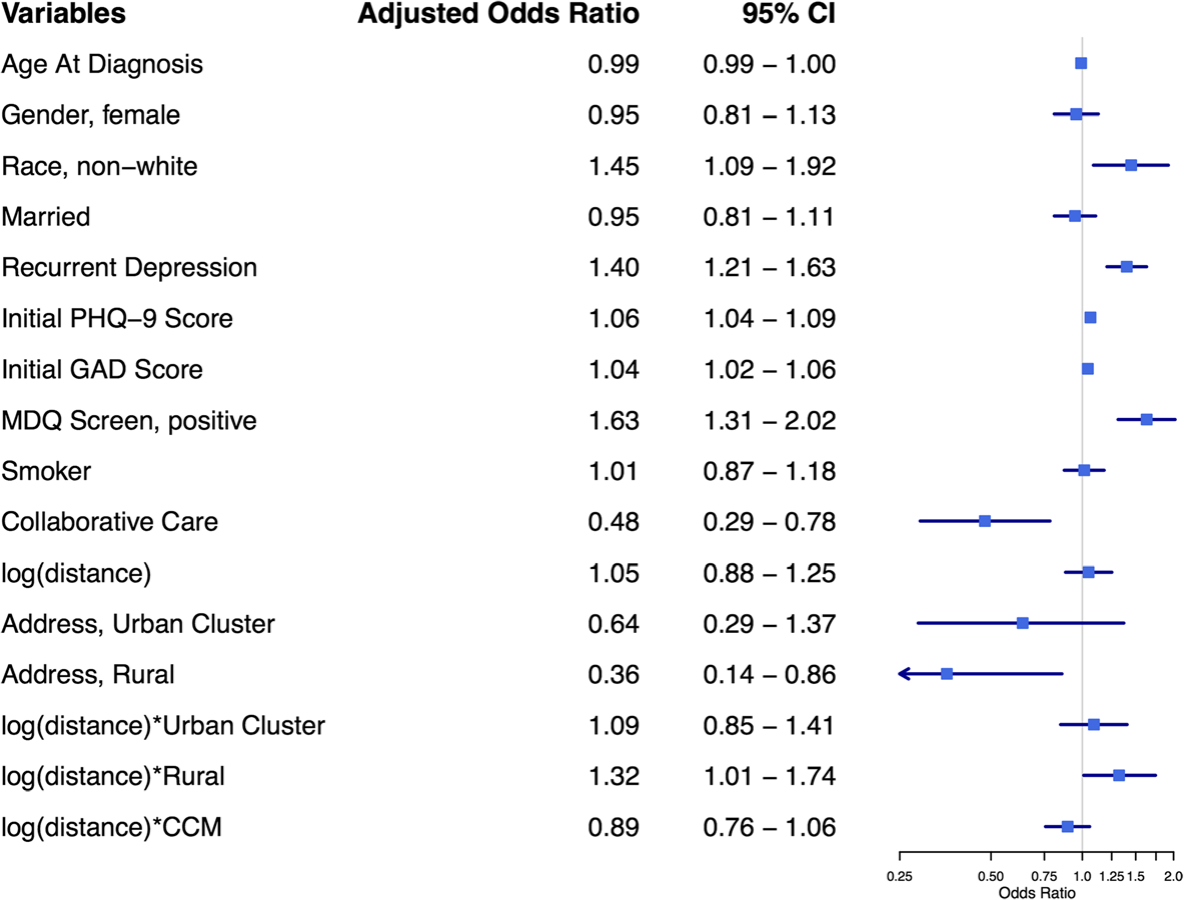
Multivariate Odds Ratios for Persistent Depressive Symptoms (PHQ-9 ≥10) at six months, controlling for clinical site. Baseline probability group is an unmarried, white, nonsmoking, male with initial depression who lives in an urban area and chooses UC. The final three lines are interaction terms between the variables separated by asterisks

The multivariate analysis of PDS reveals analogous findings as seen in Fig. 5. Rural patients had improved adjusted odds of PDS (AOR = 0.36, 95% CI 0.15–0.86) compared to urban area patients while urban cluster patients were not significantly different. The natural logarithm transformed distance to primary care clinic was associated with increased PDS for rural patients (β = 0.278, AOR 1.3, 95% CI 1.0–1.7), but not for urban area or urban cluster patients. Figure 4 demonstrates the marginal probability of PDS in a hypothetical baseline patient was lower for rural patients but increased with distance while the probability of PDS for urban area patients remained approximately constant at various distances. The marginal probability of PDS in a rural patient exceeds the marginal probability of PDS in an urban patient when the rural patient is greater than 40 km away from their primary care clinic.

Patients who were missing followup 6 month PHQ-9 values were not included in the cohort but they had the same initial depression severity (Initial PHQ-9 = 15.4 vs. 15.4, *p* = 0.347) as those in the cohort. They were younger (mean age = 39.2 yrs. vs. 42.7 yrs., *p* < 0.001), more likely to be male (29.3% vs 26.3%, *p* < 0.001), more likely to be non-white (9.6% vs. 6.6%, *p* < 0.001), less likely to be married (38.2% vs. 45.6%, *p* < 0.001), less likely to have recurrent depression (39.0% vs. 42.6%), and much less likely to have chosen CCM (41.4% vs. 79.7%, *p* < 0.001) than those in the cohort. Running a worst case analysis (*N* = 7777) that assumed lack of remission and ongoing PDS for the patients with missing 6 month PHQ-9 data yielded substantially similar results in terms of statistical significance and coefficient sign.

## Discussion

In our study of primary care patients with depression, living in a rural setting was associated with improved 6 month outcomes (remission and PDS) after controlling for other factors such as distance to clinic, demographics, marital status, enrollment in CCM, initial disease severity, and psychiatric comorbidities. This is contrary to some studies of depression which show worse outcomes in rural settings, but these did not analyze urban clusters separately nor use distance from clinic as a variable [8, 9].

Rural areas may have a greater sense of community with more ties to social groups which has been shown to be a protective factor [31]. Furthermore, our CCM model which utilizes telephone calls and online visits to supplement traditional face-to-face encounters may mitigate the reduced access to health care services traditionally seen in rural populations [32].

The improved outcomes seen in rural settings declined as the patient’s distance away from their primary care clinic increased. At 34-40 km, rural 6 month outcomes were equivalent to urban area outcomes. Distance and transportation difficulties have been identified as a major barrier to healthcare access in veterans and older rural adults [33, 34]. Additionally, almost 60% of people residing in rural areas cite the availability of high speed internet as a problem [35]. This may limit the ability of CCM to reach rural people, particularly those that reside further away from community centers where healthcare clinics are typically located. Interestingly, the distance from primary care clinic had no association with 6 month outcomes for those residing in urban clusters and urban areas, perhaps due to better transportation and telecommunications options in these communities. Future studies could specifically examine the relationship between high speed telecommunications access and depression outcomes.

CCM improved depression outcomes across all address classifications and irrespective of distance from primary care clinic. Consistent with prior studies, usual care, not being married, recurrent depression, high initial PHQ-9 score, elevated GAD-7 scores, MDQ positive scores, and current smoking status were associated with worse 6 month depression outcomes [17].

### Limitations

Although this study had a large number of subjects from multiple primary care clinics, it involves a single healthcare system with a unified model of CCM, thus limiting generalizability. Outcomes were defined by 6 month PHQ-9 scores which are commonly used in primary care, but do not necessarily reflect patient oriented outcomes such as reduced suicides, faster return to work, and improved relationships.

Furthermore, a large number of patients (5062), particularly those who choose not to enroll in CCM were excluded because they lacked 6 month outcome data. Under UC, 6 month outcome data is obtained only at the discretion of the treating clinician, even though it is a quality metric that is universally encouraged and reported [36]. There were statistically significant differences between patients having 6 month PHQ-9 outcomes and those missing followup data. Despite these known differences, the worst-case analysis did not change the statistical conclusions.

The major urban area in this study, Rochester MN, is small in comparison to other metropolitan areas across the country and may not share the racial, economic, and social diversity that exists in major metropolitan areas such as Los Angeles, Chicago, or New York. In addition, although we looked at distance from clinic, this may not correlate with travel time to clinic, especially in urban areas. Finally, we were unable to control for socioeconomic status, which can vary widely in rural areas.

## Conclusion

CCM improves the 6 month depression outcomes of remission and PDS when adjusted for confounding variables including rural vs. urban setting as defined by the US Census. Living in a rural setting is associated with improved depression outcomes but this beneficial effect declines with increasing distance between the patient’s residence and the primary care clinic.

## Data Availability

The dataset contains protected health information (such as address, dates of service). Thus, the data is available from the corresponding author for reasonable requests but may require IRB approval and data sharing agreements.

## Abbreviations

CCM: Collaborative Care Management
GAD7: Generalized Anxiety Disorder questionnaire - 7
LSAD: Legal/Statistical Area Description
MDQ: Mood Disorders Questionnaire
PDS: Persistent Depressive Symptoms
PHQ9: Patient Health Questionnaire - 9
US: United States
